# Krüppel-Like Factor 12 Promotes Colorectal Cancer Growth through Early Growth Response Protein 1

**DOI:** 10.1371/journal.pone.0159899

**Published:** 2016-07-21

**Authors:** Sun-Hee Kim, Yun-Yong Park, Sung-Nam Cho, Ofer Margalit, Dingzhi Wang, Raymond N. DuBois

**Affiliations:** 1 Departments of Melanoma Medical Oncology, The University of Texas MD Anderson Cancer Center, Houston, Texas, United States of America; 2 ASAN Institute for Life Sciences, ASAN Medical Center, Department of Medicine, University of Ulsan College of Medicine, Seoul 138–736, Korea; 3 Department of Thoracic Head and Neck Medical Oncology, The University of Texas MD Anderson Cancer Center, Houston, Texas, United States of America; 4 Biodesign Institute of Arizona State University, Tempe, Arizona, United States of America; 5 Department of Chemistry and Biochemistry, Arizona State University, Tempe, Arizona, United States of America; 6 Department of Research and Division of Gastroenterology, Mayo Clinic, Scottsdale, Arizona, United States of America; 7 Department of Biochemistry and Molecular Biology, Medical University of South Carolina, Charleston, South Carolina, United States of America; Osaka Medical Center for Cancer and Cardiovascular Diseases, JAPAN

## Abstract

Krüppel-like factor 12 (KLF12) is a transcription factor that plays a role in normal kidney development and repression of decidualization. KLF12 is frequently elevated in esophageal adenocarcinoma and has been reported to promote gastric cancer progression. Here, we examined the role of KLF12 in colorectal cancer (CRC). Indeed, KLF12 promotes tumor growth by directly activating early growth response protein 1 (EGR1). The levels of KLF12 and EGR1 correlate synergistically with a poor prognosis. These results indicate that KLF12 likely plays an important role in CRC and could serve as a potential prognostic marker and therapeutic target.

## Introduction

Colorectal cancer (CRC) is the third-leading cause of cancer deaths and the third most common cancer in the US [1]. The development of CRC depends on a series of genetic mutations and epigenetic alterations that result in progressive changes in gene expression. These changes control tumor initiation and progression. Transcription factors that regulate gene expression and certain signaling pathways during carcinogenesis are potential therapeutic targets [2], although technical difficulties usually preclude targeting them directly [3,4].

The Krüppel-like factor (KLF) family represents transcription factors that play diverse biological roles in cell differentiation, proliferation, and apoptosis by regulating specific target genes [5–7]. To date, 17 members of the KLF family have been identified in mammalian cells [8,9]. Several members of KLF family have been implicated to act as either tumor-suppressors or oncogenes in various human cancers, including CRC [8,10,11]. For example, KLF12 expression was elevated in around 40% of poorly differentiated gastric cancers (GCs) and its levels correlated with tumor size [12]. In addition, KLF12 promoted gastric cancer (GC) cell proliferation and invasion *in vitro* [12]. Recently, genome-wide analysis showed that KLF12 amplification was found in about 40% of esophageal adenocarcinoma (EAC) cases [13] and in 45% of salivary tumors [14]. However, the role of KLF12 in CRC has not been carefully addressed.

EGR1 is a key transcription factor that is involved in carcinogenesis. EGR1 has been shown to accelerate tumor growth and progression by mainly inducing cell proliferation, angiogenesis, and invasion in gastric, ovarian, prostate, and liver cancers [15–20]. On the other hand, EGR1 also exhibits a tumor suppressor function by mainly inducing tumor cell apoptosis in other types of cancers [21–25]. In CRC, EGR1 is elevated in tumors when compared to matched normal tissues [26,27] and enhances tumor cell proliferation [27–29]. However, other studies showed that activation of EGR1 induced tumor cell apoptosis [30–32]. Therefore, all of the roles of EGR1 in CRC are still not clear and may be context specific.

In this study, we show for the first time that KLF12 promotes CRC cell growth, at least in part by directly activating EGR1. Importantly, we show that KLF12 and EGR1 levels synergistically correlate with poor prognosis in CRC.

## Materials and Methods

### Cells, antibodies, and reagents

LS174T, HCT116, HT-29, SW620, LOVO and SW480 cells were purchased from the American Type Culture Collection (ATCC). Cells were maintained in McCoy 5A medium containing 10% fetal bovine serum (FBS) in a 5% CO_2_ atmosphere. Antibody to KLF12 was purchased from Santa Cruz Biotechnology (Santa Cruz, CA). Antibody to EGR1 was purchased from Cell Signaling Technology (Danvers, MA). Antibody to ACTB (*β*-actin) used to examine control protein levels were obtained from Sigma-Aldrich (St. Louis, MO). siGENOME SMARTpool siRNAs targeting EGR1 was purchased from Dharmacon, Inc. (Chicago, IL). shRNA vectors targeting KLF12 and control non-silencing vector (shCon); and KLF12 cDNA (KLF12/pLOC) and pLOC empty vector were purchased from Open Biosystems (Huntsville, AL). Lentivirus packaging vectors pMD2.G and psPAX2 were purchased from Addgene (Cambridge, MA).

### Microarray

Total RNA was extracted from cells by using a mirVana RNA isolation labeling kit (Ambion). We used 500ng of total RNA for labeling and hybridization, according to the manufacturer’s protocols (Illumina). After the bead chips were scanned with a BeadArray Reader (Illumina), the microarray data were normalized using the quantile normalization method in the Linear Models for Microarray Data (LIMMA) package in the R language environment. The expression level of each gene was log2-transformed before further analysis.

### Gene expression data of colon cancer patients

Microarray data from Moffit Cancer Center (Moffit cohort, n = 177) and Vanderbilt Medical Center (VMC) cohort (n = 55) were downloaded from GEO. Kaplan-Meier plots and log-rank test were used to estimate patient prognosis.

### Establishment of stable cell lines

pGIPZ-shKLF12, pGIPZ-shCon, KLF12/pLOC, and pLOC along with package vectors psPAX2 and pMD2.G were transfected into 293T cells in 60-mm dishes using Lipofectamine reagent (Invitrogen) according to the manufacturer's protocol. Culture medium containing virus particles was collected 48 h later and was added to target cells. Cells infected were sorted by green fluorescent protein (GFP) positivity to eliminate uninfected cells.

### DNA constructs

The EGR1 promoter (-1260 to +35) linked to the luciferase gene reporter construct was graciously provided by Dr. Eling [33]. The EGR1 promoter mutant was prepared with the QuikChange II XL Site-Directed Mutagenesis Kit (Agilent Technologies). The following primers were designed to generate the EGR1 promoter mutant: forward 5’- tggcacggtgtctttccttttttcgctgggaaattgaggataggaagtca-3’ and reverse 5’- tgacttcctatcctcaatttcccagcgaaaaaaggaaagacaccgtgcca -3’

### Luciferase assay

For dual luciferase reporter assays, cells were transfected with the firefly luciferase reporter constructs and the control renilla luciferase reporter pRL-CMV using Lipofectamine™ (Invitrogen). After treatment, cells were lysed with cell lysis buffer provided by the dual-luciferase reporter assay kit (Promega, Madison, WI). Luciferase activity was then measured according to the manufacture’s instruction.

### Cell viability assay

Ninety-six-well plates were seeded with 5,000 cells/well and cells were incubated in serum-free medium for 4 d. Cell viability was determined using PrestoBlue Cell Viability Reagent (Invitrogen).

### Western blotting

Western blot analysis was performed as previously described [34]. Total proteins were separated by loading 20μg of total cell lysate on a denaturing 10% SDS-polyacrylamide gel and transferred to a nitrocellulose membrane. Membranes were blocked with 5% non-fat dry milk and incubated with primary antibodies that recognize KLF12, EGR1, and Actin. Secondary antibody conjugated to horseradish peroxidase (Vector Laboratories Inc, Burlingame, CA) was used at 1:2,000 to detect primary antibodies and enzymatic signals were visualized by chemiluminescence. Three independent experiments were performed for all Western blotting assays.

### Real time-quantitative PCR (RT-qPCR)

Total RNA was isolated by using TRIzol (Invitrogen). cDNA was synthesized from 2 μg of total RNA by using High-Capacity cDNA Reverse Transcription Kits (Applied Biosystems, Foster City, CA) and mixed with TaqMan^®^ Gene Expression Assay Mix for KLF12 and EGR1, sterile water and TaqMan^®^ Fast Universal PCR Master Mix (Applied Biosystems). Real-time PCR was carried out using 7900 HT Fast system (Applied Biosystems) and expression of target genes mRNA relative to mRNA of beta-actin was calculated.

### Chromatin immunoprecipitation

Chromatin immunoprecipitation (ChIP) was performed using SimpleChIP Plus Enzymatic Chromatin IP Kit (Cell Signaling Technology). Briefly, cells were treated with 1% formaldehyde for 10 min at room temperature to crosslink proteins to DNA, which was then quenched by adding glycine to 0.125 M for 5 min at room temperature. Crosslinked chromatins were digested with 250 units of Micrococcal Nuclease per IP to reduce the DNA length to 150–900 base pairs. One μg of antibody was used to immunoprecipitate the crosslinked DNA per IP. After being reverse crosslinked, the DNA was purified and eluted into 50 μl of elution buffer. The amount of immunoprecipitated DNA residing in the *EGR1* promoter region was measured by Q-PCR with primers targeting *EGR1* promoter. The primers used for Q-PCR were forward 5′- cgtgacttcctatcctcaat-3′ and reverse 5′-gggcctcgatctatggcacg-3′ (site1) and forward 5′- agacctgcgggaatcgttct-3′ and reverse 5′-caaggcgagggggagaagga-3′ (site2).

### Immunohistochemistry

Human colorectal carcinoma specimens were obtained from Tissue Procurement and Banking Facility (TPBF) at The University of Texas MD Anderson Cancer Center. Paraffin-embedded specimens were treated with xylene and ethanol to remove the paraffin. The slides were immersed in Borg decloaker solution (Biocare Medical, Inc.) and boiled in a pressure cooker at 125°C for 5 min for antigen retrieval. Endogenous peroxidase activity was blocked by incubating in 3% H_2_O_2_ containing PBS solution for 10 min. The slides were blocked with 5% normal goat serum and incubated with anti-KLF12 and EGR1 antibodies at 4°C overnight. After washing with PBS, the slides were incubated with Goat anti-Rabbit HRP (Vector Laboratories). After washing, the slides were developed with DAB reagent (Vector Laboratories) followed by counterstaining with Hematoxylin.

### Animal experiments

All mice were housed and treated in accordance with protocols approved by the Institutional Animal Care and Use Committee at The University of Texas M.D. Anderson Cancer Center (IACUC Protocol No: 050706632). KLF12/LS-174T, EGR1/LS174T, GFP/LS-174T, shCTL/HCT116, shKLF12-1/HCT116, or shKLF12-2/HCT116 cells were injected into the cecal wall of athymic *nu/nu* mice. After 5 weeks post-injection mice were euthanized using CO_2_ asphyxiation. Cecal tumor weight was measured.

### Statistical analysis

Statistical significance was determined using Student's *t* test, or two-way ANOVA, where applicable. *P* < 0.05 was considered statistically significant.

## Results

### KLF12 promotes CRC growth *in vitro* and *in vivo*

**Fig 1:**
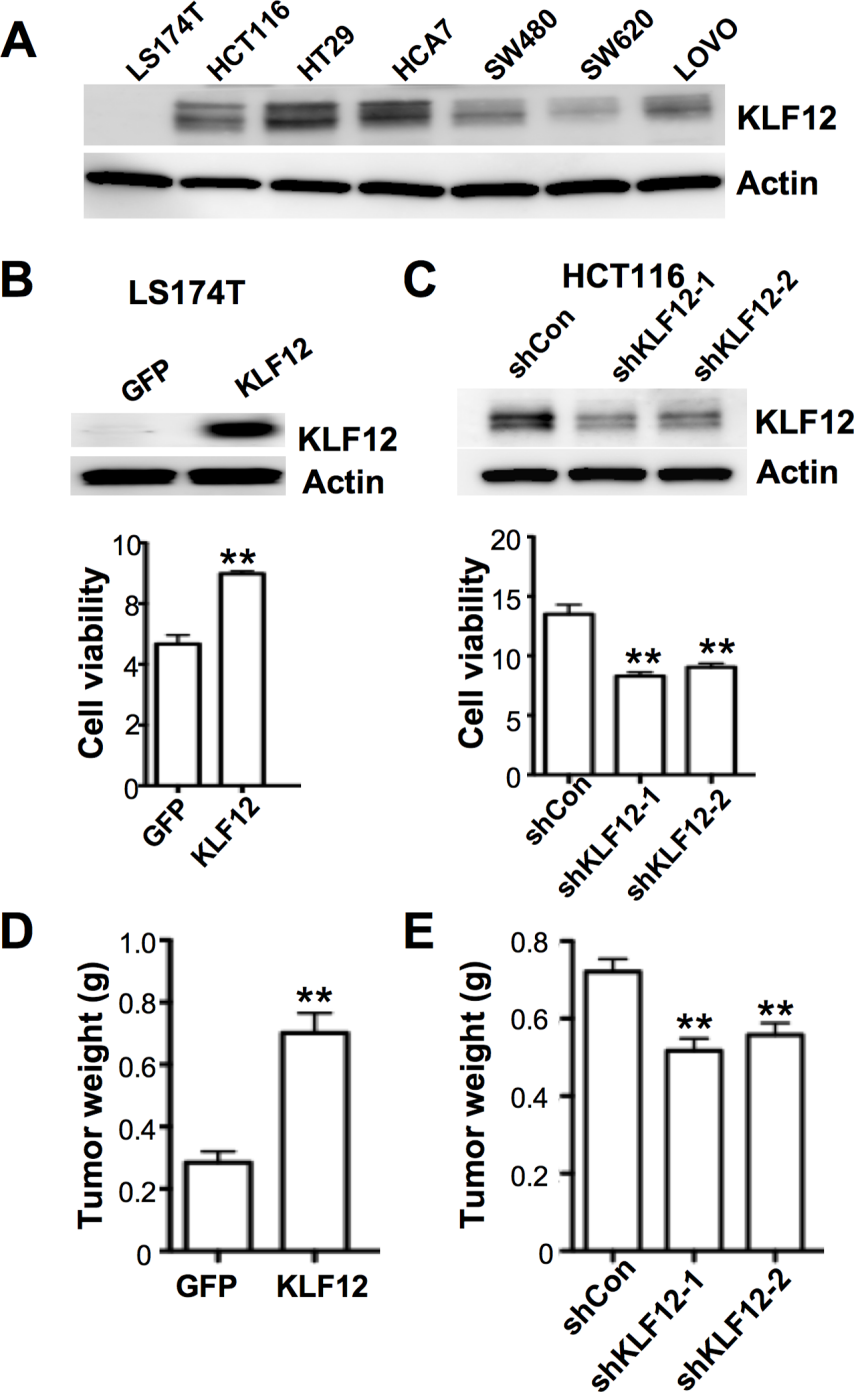
KLF12 promotes tumor growth *in vitro* and *in vivo*. (A) KLF12 protein levels in CRC cell lines. Actin served as a loading control. (B) KLF12 expression (top) and cell viability (bottom) of LS174T cells stably transfected with either GFP or KLF12. Actin served as a loading control. C. KLF12 expression (top) and cell viability (bottom) of HCT116 cells stably transfected with either a vector containing nonsilencing control shRNA (shCon) or one of two KLF12 shRNAs (shKLF12-1 and shKLF12-2). Actin served as a loading control. D. Tumor weight in mice orthotopically injected with either LS174T/GFP or LS174T/KLF12 cells (n = 8 for each group). E. Tumor weight in mice orthotopically injected with either HCT116/shCon, HCT116/shKLF12-1, or HCT116/shKLF12-2 cells (n = 9 for each group).

We first examined the levels of KLF12 in 7 human CRC cell lines and found that KLF12 was expressed in 6 of the 7 lines but not in LS-174T cells (Fig 1A). To examine the role of KLF12 in CRC cells, KLF12 was overexpressed in LS174T cells and knocked down in HCT116 and HT-29 cells. Overexpression of KLF12 led to increased cell number, whereas, knockdown of KLF12 reduced cell numbers (Fig 1B and 1C and S1A Fig). These results suggest that KLF12 enhances CRC cell proliferation and/or survival. Moreover, knockdown of KLF12 in HCT-116 cells resulted in induction of pro-apoptotic proteins such as BAX, BAK, and cleaved caspase-3 (S1B Fig), suggesting that KLF12 promotes cell survival. Furthermore, KLF12 overexpressing LS174T cells developed larger cecal tumors than vector control cells (Fig 1D), whereas KLF12 knockdown HCT116 cells formed smaller cecal tumors when compared to vector control cells (Fig 1E) in an orthotopic mouse model of CRC. Taken together, these results indicate that KLF12 promotes CRC growth.

### EGR1 is a direct target of KLF12

**Fig 2:**
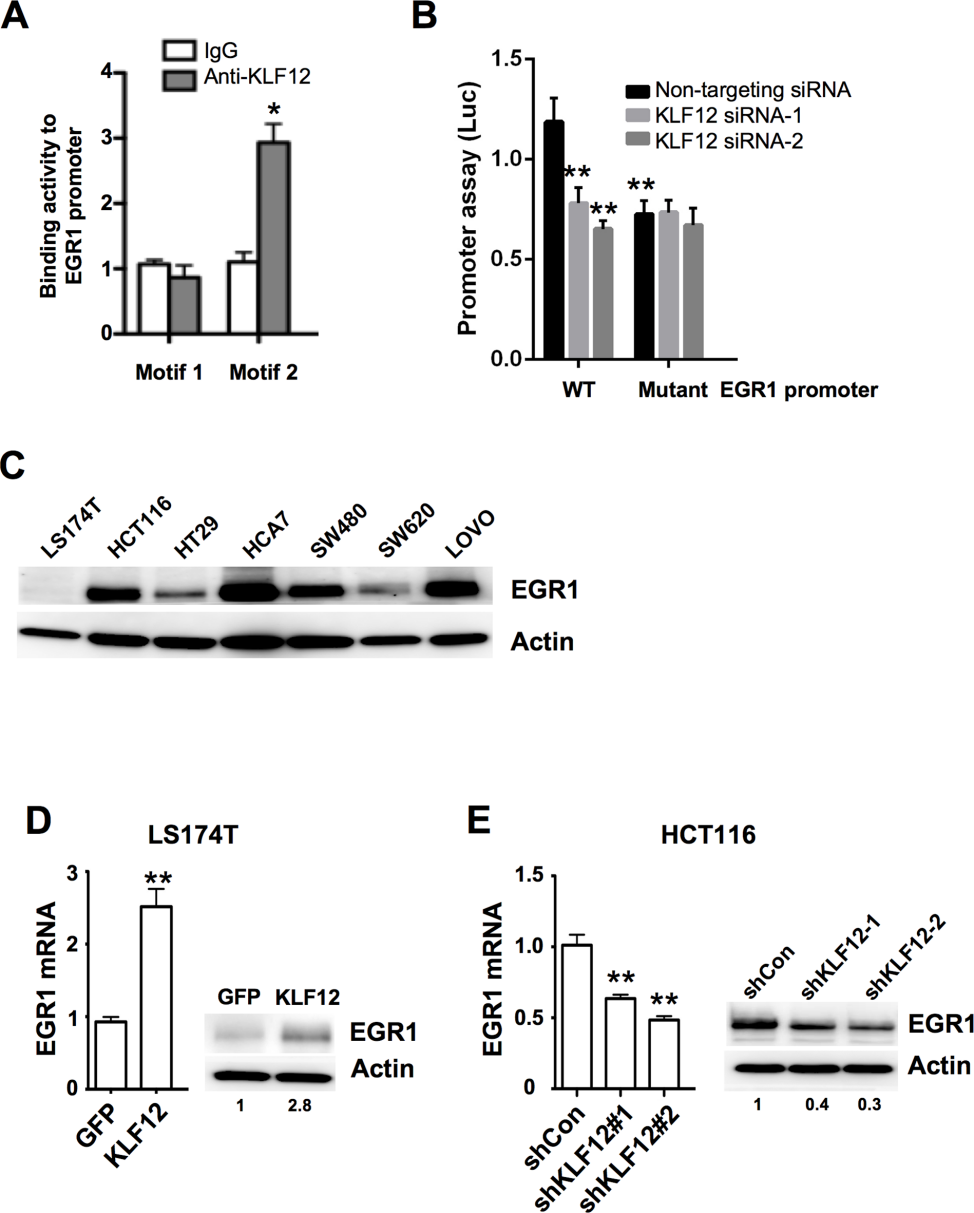
EGR1 is a direct target of KLF12 mediating cell viability. (A) Chromatin immunoprecipitation (ChIP) assay showed binding of KLF12 to motif 2 of the EGR1 promoter, but not to motif 1 in LS-174T cells. Immunoprecipitation with IgG antibody was used as a control. (B) Transient co-transfection of cells was performed with EGR1 promoter wild type (WT), or mutant luciferase reporter plasmids with renilla luciferase control plasmids and non-targeting or KLF12 siRNA. The luciferase activity was determined. (C) EGR1 protein levels in CRC cell lines. Actin served as a loading control. (D) EGR1 mRNA (left) and protein (right) levels in LS174T cells stably transfected with either GFP or KLF12. Actin served as a loading control. E. EGR1 mRNA (left) and protein (right) levels in HCT116 cells stably transfected with either a vector containing nonsilencing control shRNA (shCon) or one of two KLF12 shRNAs (shKLF12-1 and shKLF12-2). Actin served as a loading control.

**Fig 3:**
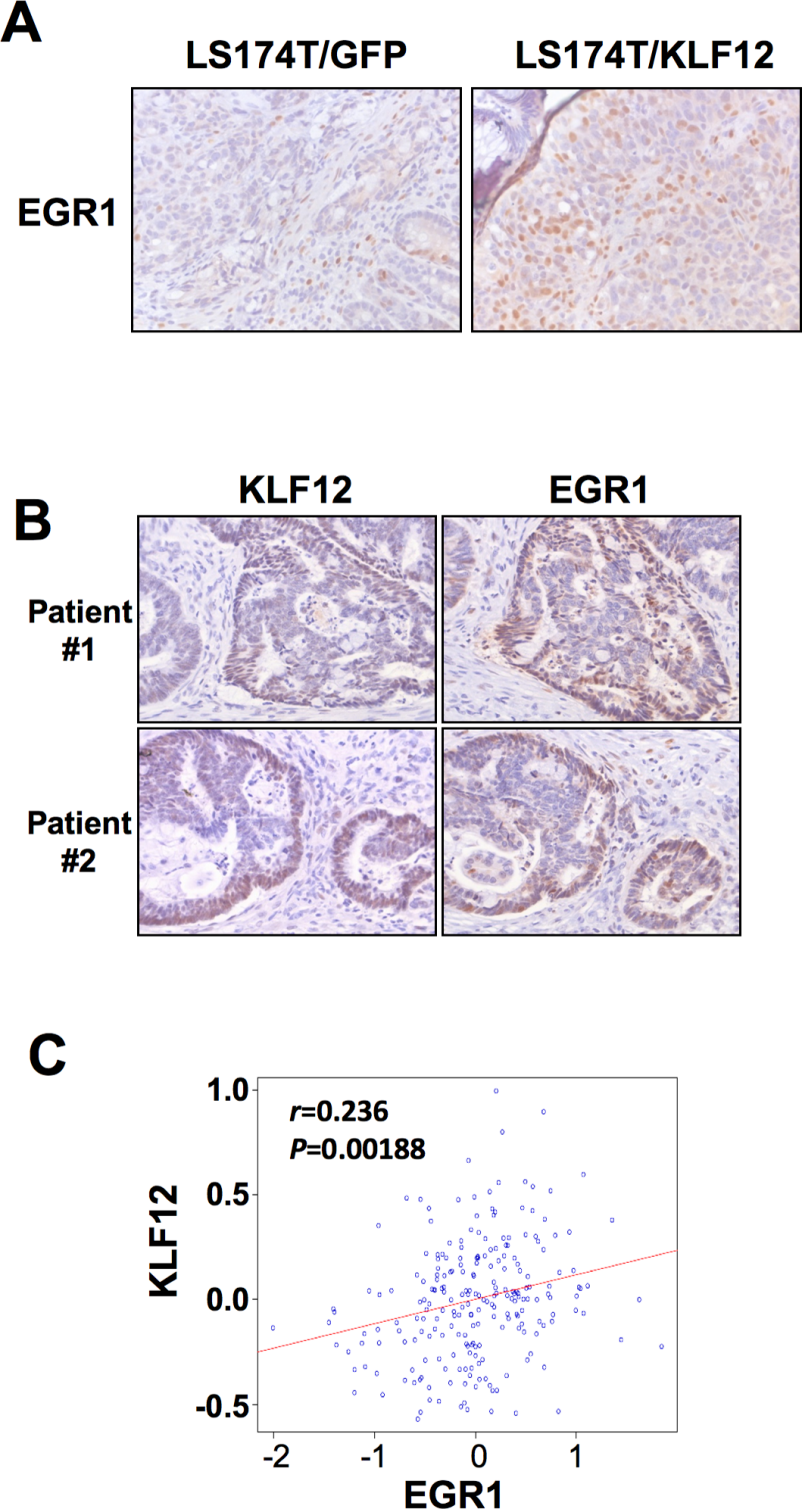
KLF12 and EGR1 is co-expressed *in vivo*. (A) Immunohistochemistry of EGR1 in nude mice injected with either LS174/GFP cells as control, or with LS174 cells stably transfected with KLF12 (LS174/KLF12). (B) Immunohistochemistry of KLF12 and EGR1 in matching sections taken from two CRC patients (Patient #1 and #2). Magnification x10. (C) Pearson correlation of KLF12 and EGR1 mRNA expression in a cohort of 232 CRC patients (Moffitt cohort, n = 177 and Vanderbilt Medical Center cohort, n = 55).

Little is known about which KLF12 target genes are involved in the regulation of CRC growth. To identify the target genes of KLF12, we performed microarray assays and found that KLF12 overexpression resulted in alteration of multiple genes, including EGR1 (S1 Table). Microarray data has been uploaded to GEO (see http://www.ncbi.nlm.nih.gov/geo/query/acc.cgi?token=ezktkiygpdcbfqb&acc=GSE78051). KLF12 has been reported to bind to the CACCC motif of target genes to regulate their expression [35]. Therefore, we first examined which above candidates have CACCC motif in their promoter regions. Interestingly, the EGR1 promoter contains two putative KLF12 DNA-binding motifs (CACCC) located at -1488bp (motif 1) and -808bp (motif 2) relative to the transcription start site [8,35]. To test whether KLF12 binds to the EGR1 promoter, we performed ChIP assay. Indeed, KLF12 strongly binds to motif 2 of the EGR1 promoter, but not to motif 1 (Fig 2A). Moreover, a mutation in the motif 2 of EGR1 promoter resulted in reduction of EGR1 promoter activity in the EGR1 promoter assays (Fig 2B). Knockdown of KLF12 reduced transcriptional activity in WT EGR1 promoter, whereas silencing of KLF-12 did not affect transcriptional activity in the mutant EGR1 promoter with a mutation in the motif 2 (Fig 2B). In addition, LS174T cells with undetectable levels of KLF12 expressed the lowest level of EGR1 protein compared with CRC cells expressing high levels of KLF12 (Figs 1A and 2C). Furthermore, overexpression of KLF12 in LS174T cells up-regulated of EGR1 expression at both the mRNA and protein levels (Fig 2D), whereas knockdown of KLF12 in HCT116 cells resulted in reduction of EGR1 expression (Fig 2E). In our animal model, EGR1 protein expression was higher in tumors isolated from mice orthotopically implanted with LS174 cells overexpressing KLF12, as compared with mice implanted with the vector control cells (Fig 3A). In human CRC specimens, KLF12 and EGR1 staining showed a similar expression pattern (Fig 3B). Finally, KLF12 mRNA levels correlated with those of EGR1 in a cohort of 232 CRC patients (Fig 3C). Taken together, these results indicate that EGR1 is directly transactivated by KLF12 in CRC.

### KLF12 enhances tumor cell growth by activating EGR1

**Fig 4:**
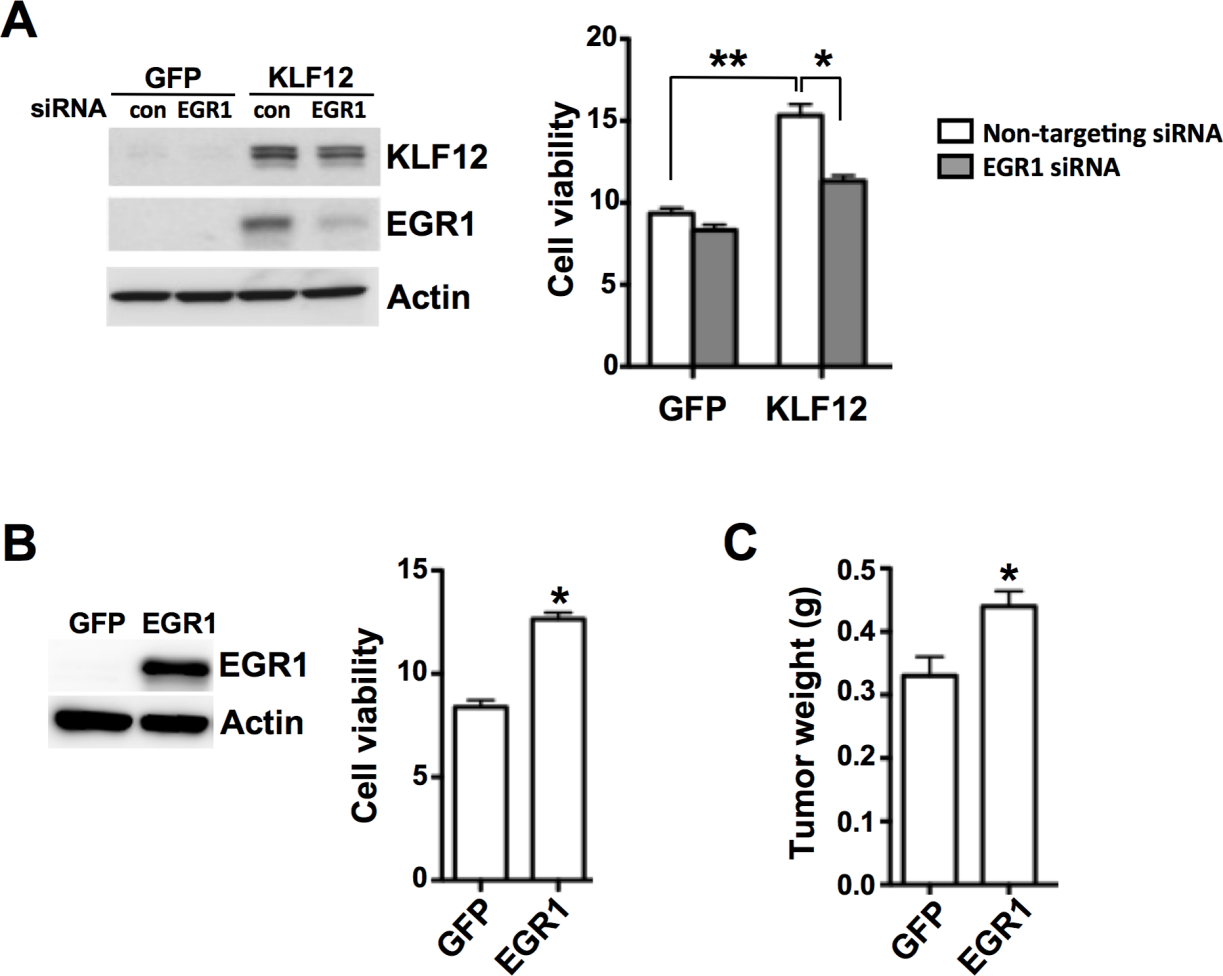
KLF12 enhances cell viability by activating EGR1. (A) Protein levels of KLF12 and EGR1 (left) and cell viability (right) of LS174T/GFP and LS174T/KLF12 cells transfected with either non-targeting siRNA as control (con) or EGR1 siRNA. Actin served as a loading control. (B) EGR1 protein levels (left) and cell viability (right) of LS174T cells transfected with either GFP (LS174T/GFP) or EGR1 (LS174T/EGR1). (C) Tumor weight in mice orthotopically injected with either LS174T/GFP or LS174T/EGR1 cells (n = 8 for each group).

To test whether EGR1 mediates the effect of KLF12 on induction of tumor cell growth, EGR1 was knocked down in KLF12-overexpressing LS174T cells. Indeed, EGR1 knockdown attenuated the KLF12-induced growth of tumor cell populations (Fig 4A). In addition, EGR1 knockdown in KLF12-overexpressing LS174T cells does not affect KLF12 expression (Fig 4A), indicating that EGR1 does not regulate KLF12. Furthermore, overexpression of EGR1 in LS174T cells promoted cell growth *in vitro* (Fig 4B) and tumor growth in our animal model (Fig 4C). Collectively, these results indicate that KLF12 enhances cell growth through activation of EGR1.

### The levels of KLF12 and EGR1 correlate synergistically with a worse prognosis in CRC patients

**Fig 5:**
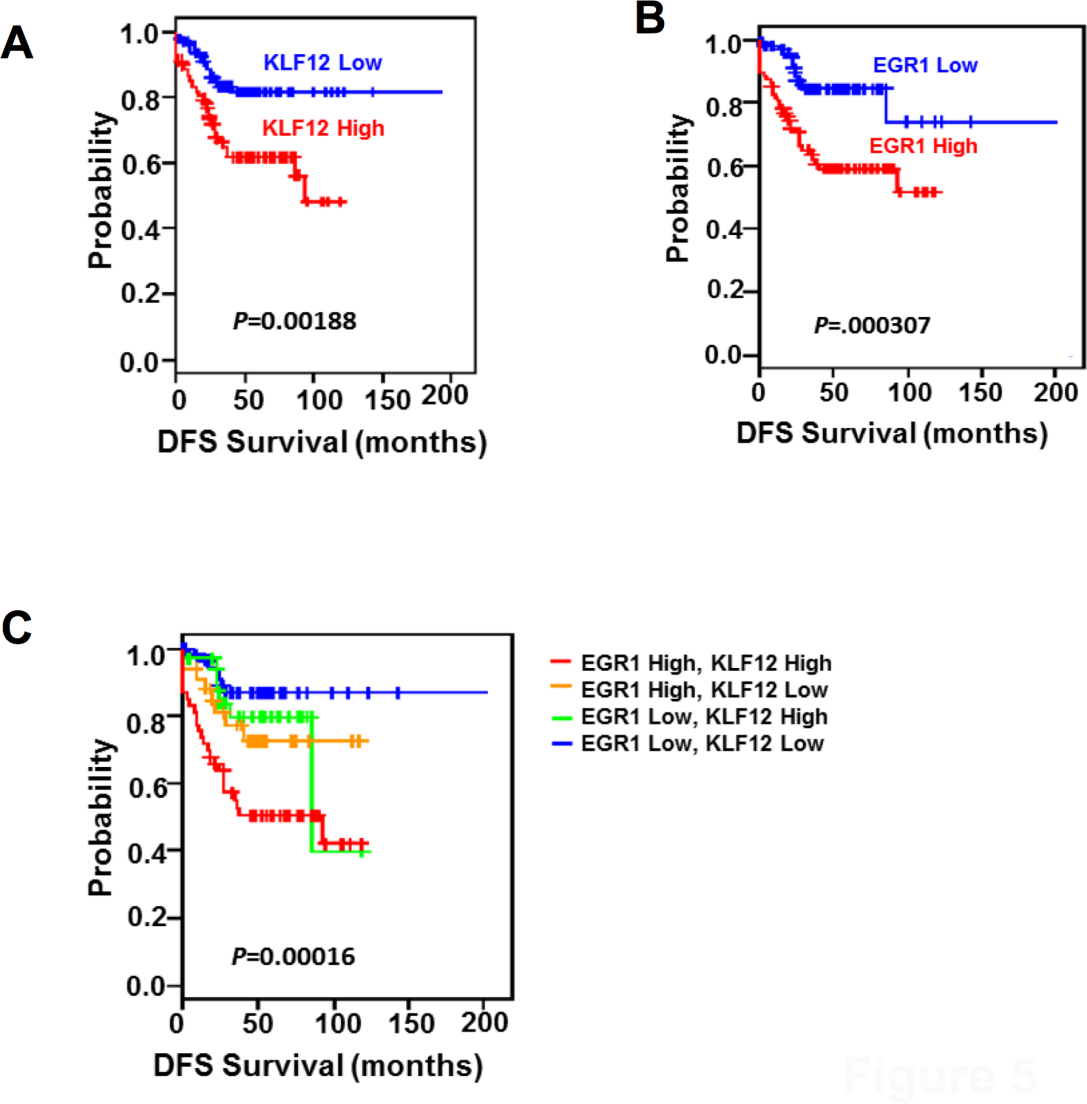
KLF12 and EGR1 expression levels are synergistically correlated with worse prognosis in CRC. Kaplan-Meier Disease free survival (DFS) curves of a cohort of 232 CRC patients (Moffitt cohort, n = 177; Vanderbilt Medical Center cohort, n = 55) with either high or low mRNA levels of KLF12 (A), EGR1 (B), or both (C). Vertical bars denote censored patients.

To further validate the clinical relevance of KLF12 and EGR1 in CRC, we evaluated whether the levels of KLF12 and EGR1 are correlated with prognosis in CRC patients. We used publicly available microarray databases to retrieve gene expression data of CRC patients. Patients in the Moffitt cohort (n = 177) and Vanderbilt Medical Center (VMC) cohort (n = 55) were dichotomized according to expression levels of KLF12 and/or EGR1. Indeed, patients with high levels of either KLF12 or EGR1 had worse outcome compared to patients with low levels of these genes (Fig 5). Importantly, patients with high levels of both KLF12 and EGR1 had the poorest survival (Fig 5C).

## Discussion

KLF12 was initially identified as a transcriptional repressor of the AP-2α gene and was suggested to mediate normal development of the kidney [35,36]. AP-2α expression is reduced in advanced CRC tumor tissues as compared to matched normal tissues [37] and loss of AP-2α promotes invasion of CRC through down-regulation of E-cadherin and up-regulation of matrix metalloproteinase 9 (MMP9) [38], indicating that KLF12 may be involved in CRC. Our results demonstrate that KLF12 promotes tumor growth in CRC. Further studies are needed to determine whether KLF12 enhances tumor cell proliferation and/or survival. In addition, our preliminary data showed that overexpression of KLF12 in LS174T cells promoted CRC cell migration and liver metastasis (data not shown). Further investigation of KLF12’s role in migration and invasion could provide better understanding of the contribution of KLF12 to CRC.

EGR1 is an early response transcription factor and its expression is very rapidly and strongly induced by growth factors, mitogens, cytokines, environmental and mechanical stresses, and DNA damage. EGR1 activates the transcription of its target genes that are involved in apoptosis, growth arrest, and stress responses [39,40]. EGR1 has been shown to provide a positive feedback loop with pro-inflammatory mediator prostaglandin E_2_ (PGE_2_) [41–48]. PGE_2_ has been demonstrated to play a major role in CRC progression [49]. In this study, we show that EGR1 is upregulated by KLF12 and mediates the effect of KLF12 on CRC cell growth, further demonstrating the role of EGR1 as an oncogene in CRC. However, there is no published data demonstrating the relationship between KLF12 and PGE_2_. Indeed, we examined whether PGE_2_ induced KLF12 expression and found that PGE_2_ did not affect the expression of KLF12 in colorectal carcinoma cells (data not shown). Moreover, we did not observe an effect of KLF12 knockdown on the COX-2 pathway (data not shown).

In summary, we provide first direct evidence that KLF12 enhances CRC cell growth, at least in part, through upregulating EGR1. Most importantly, the expression of both KLF12 and EGR1 is synergistically correlated with worse prognosis of CRC patients. Future studies are needed to not only delineate the role of KLF12 in CRC initiation, growth, and progression, but also evaluate whether KLF12 can serve as a novel prognostic marker and potential therapeutic target of CRC patients.

## Supporting Information

S1 Fig KLF12 regulates cell death in CRC cells. A. Cell viability was determined in HT-29 transfected with non-targeting or KLF12 siRNAs. B. HCT116 cells were transfected with non-targeting or KLF12 siRNAs and western blotting for BAX, BAK, and cleaved caspase 3 were conducted after 3 days.
(PDF)

S1 Table Overexpression of KLF12 alters multiple gene expression. Differences in gene expression between GFP (Control) and KLF12 overexpression (KLF12) were considered statistically significant if P < 0.001.
(XLSX)
