# Supplementary figures and images for: Krüppel-Like Factor 12 Promotes Colorectal Cancer Growth through Early Growth Response Protein 1
Source: PLoS One. 2016 Jul 21;11(7):e0159899. doi: 10.1371/journal.pone.0159899 (PMC4956169; doi:10.1371/journal.pone.0159899)

# S1 Fig

A

HT-29 cells

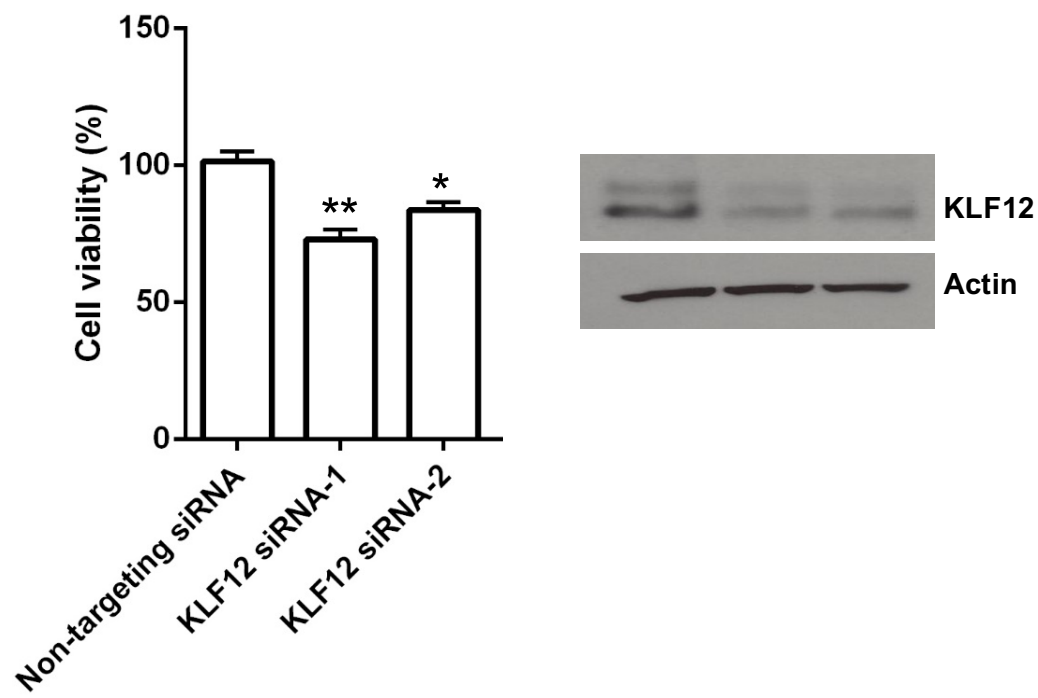

B

HCT-116 cells

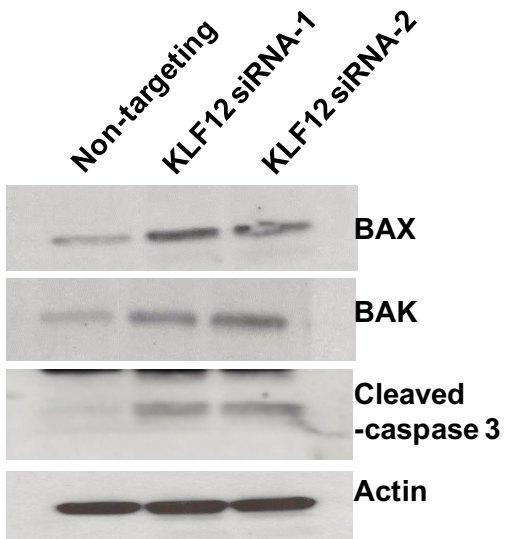

Supplement: S1 Fig — A. Cell viability was determined in HT-29 transfected with non-targeting or KLF12 siRNAs. B. HCT116 cells were transfected with non-targeting or KLF12 siRNAs and western blotting for BAX, BAK, and cleaved caspase 3 were conducted after 3 days. (PDF) [file pone.0159899.s001.pdf]
